# Supplementary material for: Computational Modeling Analysis of Kinetics of Fumarate Reductase Activity and ROS Production during Reverse Electron Transfer in Mitochondrial Respiratory Complex II
Source: Int J Mol Sci. 2023 May 5;24(9):8291. doi: 10.3390/ijms24098291 (PMC10179487; doi:10.3390/ijms24098291)
Supplement: Supplementary file 1 [file ijms-24-08291-s001.zip › ijms-2352544-supplementary.pdf]

Supplementary Data for

# Computational Modeling Analysis of Kinetics of Fumarate Reductase Activity and ROS Production during Reverse Electron Transfer in Mitochondrial Respiratory Complex II

**Nikolay I. Markevich [1,*] and Lubov N. Markevich [2]**

[1] Institute of Theoretical and Experimental Biophysics of RAS, Pushchino, Moscow 142290, Russia
[2] Institute of Cell Biophysics of RAS, Pushchino, Moscow 142290, Russia; lnmarkevich@mail.ru
* Correspondence: markevich.nick@gmail.com; Tel.: +7-9160983229

**A description of the reversible oxidoreduction of succinate, fumarate, and FAD, catalyzed by the SDH flavoprotein subunit A.**

Figure 8B in the main text presents a kinetic scheme of chemical reactions of the reversible oxidoreduction of succinate, fumarate, and FAD, catalyzed by the SDH flavoprotein subunit A (SDHA). These reactions involve the binding/dissociation of fumarate/succinate to/from the dicarboxylate binding site of the SDHA subunit as well as a single electron transfer from the iron-sulfur cluster [2Fe-2S] of the hydrophilic SDHB subunit to FAD in the SDHA subunit. The reduction of fumarate to succinate in the flavoprotein subunit SDHA shown in Figure 8B can occur via three alternative pathways. Fumarate can first bind to the dicarboxylate binding site of SDH when FAD is oxidized (reaction 20), followed by the transfer of the first (reaction 8a) and second (reaction 16a) electrons from the cluster [2Fe-2S] to FAD with the formation of the complexes $FADH^{\bullet}$.fum and FADH2.fum, respectively. In reaction 18, fumarate is reduced to succinate with the simultaneous oxidation of $FADH_2$ to FAD. Then, in reaction 19, the FAD.suc complex dissociates with the release of succinate.

In another pathway of reducing fumarate to succinate, FAD in the unoccupied dicarboxylate state is sequentially reduced first to $FADH^{\bullet}$ in reaction 8 and then to $FADH_2$ in reaction 16, receiving the first and second electrons, respectively, from the cluster [2Fe-2S]. In this case, fumarate can bind to the active site when FAD is in $FADH^{\bullet}$ (reaction 17a) or $FADH_2$ (reaction 17) state, respectively. After that, fumarate reduction to succinate and succinate release also occur as in the previous pathway (reactions 18, 19).

In addition, reactions 8b, 21a, 16b, and 21 represent the third pathway of two-electrons transfer from the cluster [2Fe-2S] to the FAD and the release of succinate when succinate is initially bound to the active center of SDH.

These three pathways of formation of succinate from fumarate, presented in Figure 8B, include four thermodynamic cycles, in which the initial and final states are identical (reactions 8a, 16a, 18, 19, 20; 16, 16a, 17, 17a; 8, 8b, 19, 21a; 8b, 16b, 17, 18, 21). Therefore, the equilibrium constants of the reactions along any cycle must satisfy the so-called “detailed balance” relationships. These detailed balance relations require the product of the equilibrium constants along a cycle to be equal to 1 because as at equilibrium, the net flux through any cycle vanishes. Therefore, such relations decrease the number of independent rate constants in a kinetic model and imply that when any one of the equilibrium constants in any cycle changes, other constants in this cycle should automatically change.

The restrictions on the kinetic constants of the reactions presented in Figure 8B are the following:

1) $K_{eq8a} \cdot K_{eq16a} \cdot K_{eq18} \cdot K_{eq19} \cdot K_{eq20} = 1$;

2) $K_{eq16a} \cdot K_{eq17a} / (K_{eq16} \cdot K_{eq17}) = 1$; (3.2)

3) $K_{eq8} \cdot K_{eq19} / (K_{eq8b} \cdot K_{eq21a}) = 1$;

4) $K_{eq8b} \cdot K_{eq16b} \cdot K_{eq17} \cdot K_{eq18} \cdot K_{eq21} = 1$.

**Mathematical model.**

A computational model corresponding to the kinetic schemes in Figure 8 and Tables 1 and 2 in the main text consists of 20 ordinary differential equations (ODE) and 7 moiety conservation equations. The model was implemented in DBSolve Optimum software, available at **http://insysbio.ru.**

Additionally, the model is presented in SBML format by separate file: Markevich_Final Reverse Scheme CII.xml as Supporting information. It should be pointed out that expressions for $d(O_2^{\cdot-})/dt$ and $d(H_2O_2)/dt$ after recover the model from SBML format have to take into account the ratio

Wimb/Wmx, where Wimb and Wmx are the fractional volume ratio of the inner membrane and matrix, respectively, to the total mitochondrial volume. See below.

*The system of ODE.* The system of ODE that was analyzed computationally in the present study can be written as follows:

$d(CII)/dt = -V_1+V_{11};$

$d(QH2)/dt = -V_1+V_{29};$

$d(CII.QH2)/dt = V_1-V_2;$

$d(CII.QH^-)/dt = V_2-V_3-V_4;$

$d([3Fe\text{-}4S])/dt = -V_3-V_5+V_6-V_{10}-V_{13}+V_{14}+V_{25};$

$d(CII.QH^\cdot)/dt = V_3+V_4-V_9;$

$d(O_2^{\cdot -})/dt = Wimb/Wmx * (V_{23}+V_{24}+V_{25}+V_{26}) – 2*V_{27};$

$d(b)/dt = -V_4+V_5-V_{12}+V_{13};$

$d(H_2O_2)/dt = Wimb/Wmx * V_{22}+V_{27}-V_{28};$

$d([4Fe\text{-}4S])/dt = -V_6+V_7-V_{14}+V_{15};$

$d(FADH2.suc)/dt = V_{16b}-V_{21};$ (1)

$d([2Fe\text{-}2S])/dt = -V_7+V_8+V_{8a}+V_{8b}-V_{15}+V_{16}+V_{16a}+V_{16b};$

$dFADH2.fum)/dt = V_{16a}+V_{17}-V_{18};$

$d(FAD)/dt = -V_8+V_{19}-V_{20}+V_{22}+V_{24};$

$d(FADH^\cdot)/dt = V_8-V_{16}-V_{17a}+V_{21a}+V_{23}-V_{24};$

$d(FAD.fum) = -V_{8a}+V_{20}$;

$d(FADH^{\cdot}.fum)/dt = V_{8a}+V_{17a}-V_{16a}$;

$d(FAD.suc)/dt = -V_{8b}+V_{18}-V_{19}$;

$d(FADH^{\cdot}.suc)/dt = V_{8b}-V_{16b}-V_{21a}$;

$d(CII.Q^{\cdot})dt = V_{9}-V_{10}-V_{12}-V_{26}$;

where the left-hand sides of the equations contain time derivatives of the concentration of various redox centers in the oxidized or reduced states, and the right-hand sides of the equations represent the rates $V_i$ (i=1, 28) of change of these variables due to electron transfer or in the process of binding/dissociation with the corresponding centers of SDH. The expressions for rates $V_i$ in SDH are presented in Table 1, and all the parameters for Ubiquinone are in Table 2 in the main text and in Supplementary Table S1 for Menaquinone. Here, the fractional volume ratio of matrix, Vmx (Wmx=Vmx/Vmit), and inner membrane, Vimb (Wimb=Vimb/Vmit), to the total mitochondrial volume, Vmit, equal approximately 0.24 and 0.652, respectively.

*Conserved moieties (in µM).* The model took into account the laws of conservation of the total concentration in the membrane of both SDH and the pools of different redox centers. These pools are presented below in the sequence, as they are presented in DBSolve Optimum software.

The total concentration of FAD centers at different states in SDH is

$Pool[1] = FAD + FAD.suc + FAD.fum + FADH^{\cdot} + FADH^{\cdot}.suc + FADH^{\cdot}.fum + FADH2 + FADH2.suc + FADH2.fum.$

The total concentration of Coenzyme Q in the inner membrane is

$Pool[2] = Q + QH2 + CII.QH2 + CII.QH^{-} + CII.QH^{\cdot} + CII.Q^{\cdot} + CII.Q$.

The total concentration of Q-binding sites at different states in complex II is

$Pool[3] = CII.Q + CII + CII.QH2 + CII.QH^{-} + CII.QH^{\cdot} + CII.Q^{\cdot}$.

The total concentration of [2Fe–2S] clusters at different oxidized and reduced states in SDH is

$Pool[4] = [2Fe–2S]^{-} + [2Fe–2S]$.

The total concentration of [4Fe–4S] clusters at different oxidized and reduced states in SDH is

$Pool[5] = [4Fe–4S]^{-} + [4Fe–4S]$.

The total concentration of cyt *b* at different oxidized and reduced states in SDH is

$Pool[6] = b^{-} + b$.

The total concentration of [3Fe–4S] clusters at different oxidized and reduced states in SDH is

$Pool[7] = [3Fe–4S]^{-} + [3Fe–4S]$.

The concentration of pools of different redox centers in SDH is taken to be equal to the total concentration of SDH. It is taken that Pool[1] = Pool[3] = Pool[4] = Pool[5] = Pool[6] = Pool[7] = 235 μM. The total concentration of coenzyme Q in the inner membrane, Pool[2], was taken to be 4541 μM, as in [11] in the main text.

*Expressions for dependent variables.* The expressions for the concentration of all seven dependent variables used in ODE are easily calculated from the pools of different variables and are presented below.

$FADH2 = - FADH2.suc - FADH2.fum – FAD – FADH^{\cdot} - FAD.fum – FADH^{\cdot}.fum – FAD.suc - FADH^{\cdot}.suc + Pool[1];$

$CII.Q = - CII – CII.QH2 – CII.QH^{-} - CII.QH^{\cdot} - CII.Q^{\cdot} + Pool[3];$

$Q = - QH2 – CII.QH2 – CII.QH^{-} - CII.QH^{\cdot} – CII.Q^{\cdot} – CII.Q + Pool[2];$

$[2Fe–2S]^{-} = - [2Fe–2S] + Pool[4];$

$[4Fe–4S]^{-} = - [4Fe–4S] + Pool[5];$

$b^{-} = - b + Pool[6];$

$[3Fe–4S]^{-} = - [3Fe–4S] + Pool[7];$

*Explicit functions.* Expressions for some important functions used in computations are presented below.

$VO_2^{\cdot-} = V_{23} + V_{24} + V_{25} + V_{26}$; the total rate of $O_2^{\cdot-}$ production by complex II in µM/s.

$VH_2O_2 = V_{22} * Wimb/Wmx + V_{27}$; the total rate of $H_2O_2$ production by the subcomplex SDHA/SDHB of SDH in µM/s expressed as WHOLE MITO Rates (Wimb=Vimb/Vmit, where Vimb and Vmit are volumes of the inner membrane and whole mitochondria, respectively) and Wimb=0.24.

It should be pointed that the stationary rate $VH_2O_2 = V_{28}$, as follows from the system of ODE (1).

*Dimension of local and whole mitochondrial concentration and rates.* Experimental data on intramembrane protein concentrations presented in Table 2 are usually presented in nmole/mg mitochondrial protein, whereas we use concentration units (µM) in our computational model. Moreover, in the model, we use local concentrations of proteins in different compartments of mitochondria, normalized by the relative volume fractions of these compartments. Therefore, matrix superoxide $O_2^{\cdot-}$ and $H_2O_2$ concentrations were normalized by the matrix water volume ($V_{MX}$), and concentrations of all intramembrane proteins of the SDHA/SDHB subunits of SDH

were normalized by the inner membrane volume ($V_{IMB}$). First, we normalized the concentration of all proteins by the total mitochondrial volume, $V_{MIT}$; then, total mitochondrial concentrations were translated into local concentrations using the water space fraction of matrix ($W_{MX}=V_{MX}/V_{MIT}$), IMS ($W_{IMS}=V_{IMS}/V_{MIT}$), and the fractional volume ratio of inner membrane ($W_{IMB}=V_{IMB}/V_{MIT}$) to the total mitochondrial volume. In order to calculate $W_{MX}$, $W_{IMS}$, and the total mitochondrial water space fraction, $W_{MITW}=V_{MITW}/V_{MIT}$, where $V_{MITW}$ is the total mitochondrial water volume, we used the following experimental data. The mitochondrial water weight fraction, $m_w/m_{mit}$, where $m_w$ and $m_{mit}$ is the mass of mitochondrial water and mitochondria, respectively, equals 0.664 g/g wet weight for a total mitochondrial density, $\rho_{mit}$, ($\rho_{mit} = m_{mit} / V_{MIT}$) of 1.09 g/ml. Because $m_w = \rho_w \cdot V_{MITW}$ and $m_{mit} = \rho_{mit} \cdot V_{MIT}$, where the water densities, $\rho_w$ and $\rho_{mit}$, are 1 and 1.09 g/ml, respectively, we can calculate the total mitochondrial water space fraction, $W_{MITW}$. Since $m_w/m_{mit} = \rho_w \cdot V_{MITW} / \rho_{mit} \cdot V_{MIT} = W_{MITW} \cdot 1\ g/ml / 1.09\ g/ml = 0.664\ g/g$, the total mitochondrial water space fraction, $W_{MITW}$, is $0.664 \cdot 1.09=0.724$.

Taking into account that $W_{IMS} \approx 1/14 \approx 0.07$ of the total mitochondrial water space for the orthodox configuration, the matrix water space fraction $W_{MX} = W_{MITW} - W_{IMS} = 0.652$. These values of $W_{IMS}$ and $W_{MX}$ are in agreement with those used in the main text. The value of $W_{IMB}$ was calculated as follows: The volume and inner membrane surface area of an average rat liver mitochondrion are 0.27 $\mu m^3$ and 6.47 $\mu m^2$, respectively. Assuming an average inner membrane thickness of about 0.01 μm, $V_{IMB}=6.47\ \mu m^2 \cdot 0.01\ \mu m = 0.0647\ \mu m^3$, the inner membrane space fraction ($W_{IMB}=V_{IMB}/V_{MIT}$) of a mitochondrion is approximately 0.24.

The mitochondrial protein weight fraction $W_{wprot}$ is about 0.25 g/g wet weight, i.e., 1 mg mitochondrial protein corresponds to 4 mg mitochondrial wet weight and occupies 4mg/1090 mg/ml = 3.67·μl. Therefore, a mitochondrial content of any metabolite of 1 nmol/mg mitochondrial protein, when normalized to total mitochondrial volume, is equal to a concentration of $10^{-9}$ mole/ $3.67 \cdot 10^{-6}$ l = 273 μM, i.e., 1 μM = 3.67 pmol/mg mitochondrial protein.

In order to present computer simulated rates of respiration and ROS production, which only occur in the inner membrane, in units of whole mitochondrial rates, we multiplied all the rates of intramembrane processes by $W_{IMB} = 0.24$.

In addition, in order to compare the computer simulated rates of respiration and ROS production presented in the current paper in μM/s with experimentally observed rates expressed in pmol/min/mg protein, the computer simulated rates can be multiplied by a factor of 3.67*60 = 220, i.e., 1 μM/s = 220 pmol/min/ mg mitochondrial protein.
